# Antibiotic resistance gradient along a large Scandinavian river influenced by wastewater treatment plants

**DOI:** 10.1093/femsec/fiag007

**Published:** 2026-02-03

**Authors:** Daniela Gómez-Martínez, Judith Sorel Ngou, Valentina Ugolini, Foon Yin Lai, R Henrik Nilsson, Erik Kristiansson, Natàlia Corcoll

**Affiliations:** Department of Biological and Environmental Sciences, University of Gothenburg, Gothenburg, Sweden; Centre for Future Chemical Risk Assessment and Management Strategies (FRAM), University of Gothenburg, Gothenburg, Sweden; Department of Biological and Environmental Sciences, University of Gothenburg, Gothenburg, Sweden; Department of Aquatic Sciences and Assessment, Swedish University of Agricultural Sciences (SLU), Uppsala, Sweden; Department of Aquatic Sciences and Assessment, Swedish University of Agricultural Sciences (SLU), Uppsala, Sweden; Department of Biological and Environmental Sciences, University of Gothenburg, Gothenburg, Sweden; Gothenburg Global Biodiversity Center (GGBC), University of Gothenburg, Gothenburg, Sweden; Centre for Future Chemical Risk Assessment and Management Strategies (FRAM), University of Gothenburg, Gothenburg, Sweden; Department of Mathematical Sciences, Chalmers University of Technology and University of Gothenburg, Gothenburg, Sweden; Department of Biological and Environmental Sciences, University of Gothenburg, Gothenburg, Sweden; Centre for Future Chemical Risk Assessment and Management Strategies (FRAM), University of Gothenburg, Gothenburg, Sweden; Gothenburg Global Biodiversity Center (GGBC), University of Gothenburg, Gothenburg, Sweden

**Keywords:** antibiotic resistance, sediment, WWTP, qPCR, shotgun metagenomics

## Abstract

Recent studies have identified the environment as a key reservoir from which antibiotic resistance genes (ARGs) can be acquired and transmitted to pathogens. However, our knowledge about the presence of ARGs in high-flow river sediments is still limited. We analyzed the resistome of sediment bacterial communities along the Swedish river Göta Älv and investigated the potential dissemination of ARGs and antimicrobials from effluents of wastewater treatment plants (WWTPs). While we detected nine different antimicrobials in the effluent water from the WWTPs through HPLC-MS, their presence was not observed in the river surface water. Analysis by qPCR revealed that the genes *sul1* and *ermB* were the most dominant ARGs among sediment, sludge, and effluent samples. Shotgun metagenomics revealed unique differences between the sludge resistomes of the WWTPs. Moreover, our findings show that ARGs increase downstream of the Göta Älv and their diversity differs from that of the upstream sites. Efflux pump resistance-related genes were most abundant in sediment samples, and beta-lactams and tetracyclines were the most common antibiotic classes targeted by ARGs. Our study emphasizes the importance of urban river sediments as a reservoir of ARGs, as tracking ARGs in WWTPs and their receiving environments improves our understanding of their spread and characteristics.

## Teaser

We characterized the resistome of sediment bacteria along the Swedish river Göta Älv and investigated the potential dissemination of ARGs and antimicrobials from the effluents of its associated wastewater treatment plants (WWTPs).

## Introduction

Antibiotic resistance (AR), defined as the mechanism by which bacteria increase their tolerance against antibiotic exposure (Christaki et al. 2020; WHO 2017), has become one of the most significant public health threats of the 21st century (Tang et al. 2023). Genes encoding biochemical mechanisms for this trait are known as antibiotic resistance genes (ARGs) and are often transferred horizontally between bacterial cells. The worldwide high usage of antibiotics for human and veterinary purposes has induced a higher abundance of AR bacteria in the gut microbiome (Zeng et al. 2019), resulting in a higher release of ARGs to the environment (Mokracka et al. 2012, Osińska et al. 2020). In addition, the role of the environment as a key source and dissemination route of resistance has been increasingly acknowledged (Bengtsson-Palme et al. 2018). River sediment has been recognized as a reservoir of ARGs (Lu et al. 2010, Calero-Cáceres et al. 2017, Zhang et al. 2022), and the characterization of its resistome is of importance to understand which ARGs are maintained by environmental communities, and how they spread between environmental and host-associated bacteria (Leonard et al. 2015).

Urban wastewater treatment plants (WWTPs) have been suggested as hotspots for antibiotic residues and ARGs (Bengtsson-Palme et al. 2018, Drane et al. 2024; J. Guo et al. 2017, Osińska et al. 2019, Rizzo et al. 2013). Until recently, the spread of antimicrobial resistance (AMR) in treated wastewater has not been considered in regulation and monitoring (Björkman et al. 2022), and most of the WWTPs are not capable of fully removing antibiotic-resistant bacteria (ARB), ARGs, and antibiotic residues before discharging the effluent into the receiving aquatic environment.

In response to the AMR crisis, in 2015, the World Health Organization (WHO) published a Global Action Plan on AMR, embracing a One Health approach (animals, humans, and environment), where member states were asked to develop and implement national action plans based on the global action plan objectives (Björkman et al. 2022). Since then, there has been an increasing awareness on directing more attention towards the occurrence, spread, and risk of AMR in environmental compartments (Liguori et al. 2022). During the last few years, Sweden has faced an increase in several problematic types of ARBs, in particular carbapenem resistance in Gram-negative bacteria (Swedres-Svarm 2023). It is known that even low abundances of ARGs and ARB can contribute to the persistence and spread of antibiotic resistance within environmental bacterial communities (Lin et al. 2021, Sánchez-Baena et al. 2021). Therefore, screening of ARGs in WWTPs and their receiving environment deepens our understanding of their dissemination and helps to guide management actions (Grenni 2022, Liguori et al. 2022, Wang et al. 2024). Furthermore, studies have shown that resistant bacteria can be reintroduced into the human microbiome via AMR- and ARG-polluted waters through, e.g. ingestion of sewage-contaminated water during recreational activities, consumption of crops irrigated with surface water, or through other contexts of poor sanitation (Bengtsson-Palme et al. 2023, O’Flaherty et al. 2018).

Sediments harbor the highest microbial diversity in aquatic environments and consequently could serve as ARG reservoirs (Lu et al. 2010). Thus, it is reasonable to deduce that a wide variety of ARB might exist in sediment environments, as they are taxon-rich habitats, particularly so for sediments receiving WWTP effluent water. Such diverse microbial communities are expected to contain resistance genes beyond those typically found in the human microbiome, thereby representing potential sources for the recruitment of ARGs into pathogenic bacteria (Pal et al. 2016). Moreover, ARGs can persist for long periods in sediments and may be more available during high flow events due to resuspension into the water (Calero-Cáceres et al. 2017). Studies in low and moderate-flow rivers where the discharges from WWTP effluents into the river represent a significant percentage of the river flow (e.g. Mediterranean rivers) have been relatively more studied (Aujoulat et al. 2021, Marizzi del Olmo et al. 2025, Pantanella et al. 2020) than rivers with high-flow, where the discharges from WWTP effluents into the river represent a lower proportion of the total river flow.

The Göta Älv is a 96-km high-flow Scandinavian river that receives wastewater discharges from four municipalities, including Gothenburg’s WWTP and has served as a raw water source for Gothenburg (the second largest city in Sweden) and surrounding towns since the late 19th century. While concentrations of other pollutants in the river water have generally met drinking water standards, faecal contamination remains a persistent and significant risk. This threat is intensified during flood and heavy rain events. During such events, the sewage infrastructure is unable to cope with such high volumes of water, and the first flush of storm water bypasses the WWTPs. In this region, floods are driven by both high precipitation and elevated sea water levels, creating recurrent pressures on the river. In this context, the present study provides the first description of the resistome in the sediments of the Göta Älv. This river is not only critical for regional water supply but is also representative of high-flow boreal rivers. By characterizing the resistome in this river, we address a significant knowledge gap with both local and broader environmental and clinical relevance.

Current antibiotic resistance (AR) surveys are mainly based on culturing pathogens and bacterial indicators (Abramova et al. 2023, Anjum et al. 2021). However, these methods are often unsuitable for tracking AR in the environment, since most environmental bacteria are hard to isolate and culture under laboratory conditions. Modern molecular methods, such as high-throughput-sequencing (metagenomics) and real-time quantitative PCR (qPCR), have largely sidestepped these limitations. Metagenomics is a nontargeted method that allows a comprehensive profiling of ARGs in environmental samples, providing information on the most common ARGs within the sample. However, it is a semiquantitative method and has a limited sensitivity for detecting rare, uncommon genes. qPCR, on the other hand, provides highly sensitive and consistent detection and quantification of ARGs, but its utility relies on selecting the right target genes and defining suitable reference values (Abramova et al. 2023, Larsson and Flach 2022).

In this field study, we hypothesized that ARGs accumulate downstream in the river and that the resistome in the downstream regions might differ from those of the upstream regions, after the river has taken WWTP effluents and other runoff waters that might add ARGs to the river load. Therefore, we aimed to characterize the presence of ARGs within sediment bacterial communities along the Göta Älv, by using and comparing the two most common culture-independent methods for this purpose: qPCR and shotgun metagenomics. We also aimed to assess if WWTP discharges serve as sources of antimicrobials and ARGs into the Göta Älv, as well as to characterize and link the bacterial diversity of river sediment with its resistome. To do so, we quantified a set of six mobile and commonly found ARGs of clinical importance (i.e. *bla*_CTX-M_*, ermB, qnrS, sul1, tetA*, and *tetX*) via qPCR in the four WWTPs and the river sediment along the Göta Älv. We furthermore performed shotgun metagenomics to obtain a broad profiling of the resistome and identify the most common ARGs in the Göta Älv and its associated WWTPs by analyzing the resistome in the effluents and sludge, which were used as references. In addition, we characterized the river in terms of antimicrobial residues by analyzing water from each WWTP effluent and along the river surface water.

## Materials and methods

### The study site

The study was conducted in the river Göta Älv, a ca. 96-km boreal river originating in Lake Vänern (the largest lake in the European Union), and running into the Kattegat Sea, by the city of Gothenburg, located on the Atlantic Swedish west coast. Nowadays, it has the largest catchment area in Scandinavia (50 000 km^2^), with an average flow rate of 575 m^3^/s. The total height difference down to sea level is 44 m. The river is the source of supply for drinking water for the city of Gothenburg (the second largest city in Sweden) with more than one million inhabitants including the metropolitan area and other small cities in the catchment. The Port of Gothenburg, located in the Göta Älv estuary, is the largest and most important port in Scandinavia. The Göta Älv catchment and the city of Gothenburg have a long history of anthropogenic activities such as hydropower plants and locks, shipping, industry, infrastructure including large roads and railroads, settlements, and discharges from WWTP effluent-waters of four municipalities, including the discharge from Gothenburg’s WWTP. All four WWTPs that discharge their effluent waters into the Göta Älv river/estuary were included in this study. From headwaters into the estuarine zone, they are WWTP of Vänersborg (39 748 users, 1.5 km), WWTP of Trollhättan (59 154 users, 17 km), WWTP of Lilla Edet (6464 users, 50 km), and the WWTP of Gothenburg (887 442 users, 97 km). The sampling was done between the 3rd and the 6th of October 2022. More specifically, samples from Vänersborg were collected on the 3rd, those from Trollhättan on the 4th, those from Lilla Edet on the 5th, and those from Gothenburg on the 6th of October. Characteristics of each WWTP are described in Table S1. A total of seven sites were sampled along the Göta Älv. Each site was located around 1 km upstream or 1 km downstream of the outlets of the four WWTPs (Fig. S1). The site downstream of the WWTP in Gothenburg was not included in the present study, since the effluent is emitted in the marine part of the estuary. The coordinates and general water quality parameters of each sampling site are provided in Table S2.

### Sludge and effluent water sampling

In all WWTPs, sludge and effluent samples were collected in triplicate. Sludge samples for DNA analysis were collected from the aeration tank and placed into sterile 50-ml Falcon tubes. In the lab, the sludge was homogenized and aliquoted into different preweighed 2-ml Eppendorf tubes for DNA extraction. All samples were centrifuged at 8000 g for 10 min. The supernatants were removed before storing the samples at −80°C until DNA extraction.

Effluent samples for antimicrobial analysis were collected in polypropylene (PP) bottles that were previously rinsed with methanol (MeOH) 100% and MilliQ water. Water samples for DNA analysis were collected in previously autoclaved glass bottles (500 ml of effluent water). All the samples were transported on ice to the laboratory within 4–6 h after sampling. For antimicrobial analysis, 200 ml of effluent water were filtered through 0.45-µm nitrate cellulose membrane filters and stored at −20°C. For the DNA analysis of effluent samples, the effluent water was subsequently filtered through GF/F (0.7 µm), 0.45 µm, and 0.2-µm nitrate cellulose membrane filters. The 0.2-µm filter papers were folded with sterile forceps and stored in 2-ml Eppendorf tubes at −80°C until DNA extraction.

### River sediment sampling

In all river sites, water and sediment samples were collected on the same dates as the samples from the WWTPs (see section, The study site). Sediment samples were collected at the riverbanks (depth < 1 m) of each sampling site using a bottom sediment hand core sampler (length 50 cm, diameter 5.2 cm; SWEDAQ, Sweden). The top 2 cm of the sediment core were transferred to sterile 50-ml Falcon tubes with the help of a sterile spoon. The Falcon tubes were filled up to the 30-ml mark. Sediment samples were collected in triplicates in each sampling location, and three cores were used for each replicate. The samples were stored in the dark and on ice for transportation to the laboratory within 4–6 h after sampling. In the laboratory, the sediment was homogenized with the help of a sterile spatula and then aliquoted into different preweighted tubes for further biological analyses: 15-ml Falcon tubes for chlorophyll-*a* content determination (Table S2), 20-ml glass scintillation vials for dry weight (DW) determination, and 2-ml Eppendorf tubes for DNA extraction. The samples for DNA analyses were centrifuged at 8000 g for 10 min, and the supernatant was removed before storage at −80°C.

### Sediment characterization

Dry weight (DW) was used as an estimate of total biomass in the sediment. For its determination, the frozen sediment samples weighed, vacuum-dried for 24 h, and reweighed. For Chl-*a* analysis, the frozen sediment samples were vacuum-dried for 24 h and pigments were extracted and identified following Gómez-Martínez et al. (2024). The content of chlorophyll-a (Chl-*a*) was used as a proxy for the total algal biomass and is expressed in micrograms of chlorophyll-a/gDW (Table S2).

### Surface water sampling

General physicochemical parameters of water quality, nutrients, and antimicrobial levels were characterized for the surface water in all sampling sites. *In situ* physicochemical parameters (i.e. pH, temperature, and conductivity) were collected using a multiparameter instrument (HANNA Instruments, Italy). For nutrient and dissolved organic carbon (DOC) analysis, a total of 45 ml of surface water was stored in 50-ml sterile Falcon tubes. For antimicrobial quantification, a total of 200 ml of surface water was collected and stored in prelabeled PP bottles that were previously rinsed with MeOH 100% and MilliQ water. All samples were taken in triplicate and stored in dark and cold conditions for transportation to the lab, where they were stored at −20°C until further analysis. Nutrient analyses were performed by Eurofins Water Testing Sweden using standard spectrophotometric methods (phosphate: ISO 15923-1: 2013 Annex F, nitrate: ISO 15923-1: 2013 Annex F, and DOC: SSEN 1484: 1997). Values for general water quality parameters are provided in Table S2.

### Antimicrobial chemical analysis of water samples

A total of 30 antimicrobial chemicals were screened in WWTP effluent water (40 ml) and surface water (200 ml) upstream and downstream of the WWTPs, based on a previously validated method (Ugolini and Lai 2024). Briefly, solid-phase extraction was performed with Oasis WCX cartridges (150 mg, 6cc) on filtered samples, with previous adjustment to pH 6, addition of Na_2_EDTA, and spiking of internal standards (IS) mixture (250 ng/L for effluent water; 50 ng/L for surface water). After elution (5 ml MeOH + 5 ml 4% FA MeOH), preconcentration under nitrogen flow (to 20 µL), and reconstitution, the SPE extracts (200 µL) were analyzed together with ten calibration standards (0–200 ng/mL, IS 50 ng/mL) using ultra-high performance liquid chromatography coupled to tandem mass spectrometry (Exion^®^ LC, Sciex^®^ Triple-Quad 3500). The method’s quantification limit ranges were 2.5–470 ng/L in WWTP effluent water and 0.5–75 ng/L in surface water. Within-run precision (relative standard deviation) for the quantified chemicals was <25% (range 0.92–10%) with only clarithromycin showing a higher variation (29%) (Table S5). Recovery of the chemicals was in the range of 52–131% in WWTP effluent water and 46–136% in surface water (Table S5). MilliQ water blank samples showed no contamination with the target compounds.

### Genomic DNA extractions

The bacterial genomic DNA from the sediment and sludge samples, as well as the frozen filters from WWTP effluent water, were extracted using the Fast DNA SPIN Kit for Soil (MP, Biomedicals, USA) according to the manufacturer’s instructions. To avoid contamination, the DNA extractions were performed under sterile conditions. DNA quality data are provided in Table S6. The concentration and purity of the extracted DNA were determined using a Qubit fluorometer and a Nanodrop Spectrophotometer, respectively. Clean DNA extracts were stored at –20°C and subsequently used for qPCR and metagenomics analyses.

### Analysis of ARGs using qPCR

Quantitative polymerase chain reaction (qPCR) was used to amplify and quantify the targeted mobile ARGs (*bla*_CTX-M_*, ermB, qnrS, sul1, tetA*, and *tetX)* and one reference gene *(rpoB*) from the extracted DNA samples (see primer selection in Table S3). The ARG gene primers have all been previously used in wastewater contexts. The analyses were performed using SYBR green detection chemistry on a CFX96 Touch real-time PCR machine, using the CFX Maestro software v. 2.0 (BIO RAD, USA). Each gene was amplified using specific primer sets obtained from the literature and qPCR conditions optimized during this study (Table S4). Three technical replicates were averaged for each sample. A dissociation curve was applied at the end of each run to detect nonspecific amplifications. Five-fold serial dilutions of the standards for each gene were run in parallel with DNA samples, no-template controls (qPCR premix without DNA template), and positive controls. Five-fold serial dilutions of DNA containing known concentrations of the target gene were used as standard curves, which were generated by quantifying the DNA from amplicons of positive controls (sludge samples) using a Qubit fluorometer. PCR amplicons for all target genes were sequenced by Sanger sequencing to confirm their identity. The FASTA sequences were identified using the BLAST tool on the CARD (Comprehensive Antibiotic Resistance Database) website (https://card.mcmaster.ca/analyze/blast) to confirm the specificity of the qPCR primers and the identity of the amplicon sequences.

### Univariate statistical analysis and Pearson correlations

Statistical differences in qPCR-quantified ARGs among the WWTP sludge, effluents, and river sediment samples were assessed through one-way analysis of variance. The obtained *P*-values were adjusted by a Tukey post-hoc test for multiple comparisons using the R package multcompView v 0.1–10 (Graves et al. 2024). To compare the results obtained from qPCR and shotgun metagenomics (see section, ARGs characterization via DNA shotgun metagenomics), Pearson correlations were performed between the total number of *sul1* and *ermB* copies obtained in qPCR and the total number of *sul1* and *ermB* counts obtained in the shotgun metagenomics samples (the rest of the genes were not found in river samples using shotgun metagenomics). Moreover, Pearson correlations were also performed to assess the link between the resistome and the effluents, distance from the river source, and number of users on each WWTP using the function corr.test() in R v.4.2.1 for pairwise data. In all tests, *P*-values < 0.05 were considered statistically significant.

### ARGs characterization via DNA shotgun metagenomics

#### Sequencing

Samples corresponding to the WWTP sludge from Trollhättan, Lilla Edet, and Gothenburg (three samples), and river sediment samples (seven samples) from upstream and downstream, and the four WWTPs were sequenced. Three replicates of each sample were pooled into a joint sample prior to DNA sequencing. Due to a DNA amount limitation, it was not possible to sequence the sludge sample from the WWTP in Vänersborg and the effluent samples of all WWTPs. One sequencing library from each of the 10 DNA samples was produced using the TruSeq PCRfree DNA library preparation kit (Illumina Inc.). Cluster generation and 150 cycles paired-end sequencing of the 10 libraries were performed in one lane of an SP flowcell using the NovaSeq 6000 system and v1.5 sequencing chemistry (Illumina Inc.). Each sample generated at least 38.4 M reads. Sequencing was performed by the SNP&SEQ Technology Platform in Uppsala. Raw sequences were deposited to the NCBI sequence read archive with BioProject accession number PRJNA1264511 (https://www.ncbi.nlm.nih.gov/sra/PRJNA1264511).

#### ARG screening and taxonomic profiling

The quality of paired-end, 100 bp long raw reads was evaluated using fastQC v 0.11.9 (https://www.bioinformatics.babraham.ac.uk/projects/fastqc/). The sequences were trimmed for quality and adapter contamination using BBDuk v 38.90 (https://github.com/BioInfoTools/BBMap/blob/master/sh/bbduk.sh) with the following parameters: ref=adapters.fa ktrim=r k=23 mink=11 hdist=1 tpe tbo qtrim=r trimq=10. Subsequently, the DNA sequences were blasted against protein sequences (blastx) in the Comprehensive Antibiotic Resistance Database (CARD; Alcock et al. (2023) using diamond v.2.1.9 (Buchfink et al. 2021). The obtained hits were filtered based on a minimum identity cutoff of 90% and an alignment length threshold of 75 bp (25 aa), removing alignments corresponding only to conserved regions between ARGs and non-ARGs, while retaining genuine ARG sequences. When a read aligned well to several high-quality ARGs, the best hit for each read was selected. If two or more hits were equally good, one of them was selected at random. Finally, a count matrix was obtained for each sample and ARG. Genes that confer resistance by nonmobile mutations were excluded from further analysis. The final gene counts (156 ARGs) were normalized to the total bacterial counts in each sample obtained from metagenomic taxonomic assignments using Kraken2 v.2.1.2 (Wood et al. 2019).

The ARG profiles were also grouped according to the two CARD ontology terms (Antibiotic Resistance Ontology: ARO) “antibiotic class” and “resistance mechanism.” Differentially abundant ARGs among sediment samples were identified using the DESeq2 v 1.36.0 package (Love et al. 2014), from Bioconductor v 3.15 (https://bioconductor.org/packages/release/bioc/html/DESeq2.html), through a likelihood ratio test to evaluate the expression changes across more than two levels (each kilometer of the river was set as a level). The resulting *P*-values were adjusted using the Benjamini–Hochberg false discovery rate (FDR) algorithm. Differentially abundant ARGs along the river segments were considered significant using an FDR cut-off of 0.1.

Already quality-trimmed sequences were classified to all taxonomic levels using Kraken2 v. 2.1.2 with the prebuilt standard Kraken2 database (released on 06/05/2023). Relative abundances were obtained with Bracken v2.6 (Bayesian re-estimation of abundance after classification with Kraken; Lu et al. 2022), which estimates abundances at multiple taxonomic levels (i.e. genus and class). Taxonomic annotations that did not comprise at least 0.001 of the total fraction of reads were excluded from further analysis. All plots were generated using the ggplot2 v3.5.1 package in R.

**Table 1:** Shannon index values in sludge and river sediments along the Göta Älv, at the genus level.

Sample ID	Sampling site	Shannon index (genus)
up_V_WWTP	upstream Vänersborg WWTP	4.41
dw_V_WWTP	downstream Vänersborg WWTP	4.54
up_T_WWTP	upstream Trollhättan WWTP	4.57
dw_T_WWTP	downstream Trollhättan WWTP	4.51
up_L_WWTP	upstream Lilla Edet WWTP	4.66
dw_L_WWTP	downstream Lilla Edet WWTP	4.52
up_G_WWTP	upstream Gothenburg WWTP	4.14
s_T_WWTP	sludge Trollhättan WWTP	4.38
s_L_WWTP	sludge Lilla Edet WWTP	4.49
s_G_WWTP	sludge Gothenburg WWTP	4.28

#### crAssphage alignment

All metagenomes were scanned for crAssphage presence as an indicator of human faecal pollution following Karkman et al. (2019). Briefly, the NCBI reference sequence for crAssphage (*Carjivirus communis*) NC_024711.1 was retrieved and indexed. The metagenomes were also indexed and mapped against the crAssphage reference genome using Bowtie2 (v2.3.0) paired-end alignment default parameters and samtools (v.1.6) to convert the output SAM files into sorted BAM files. The average genome coverage was used as a proxy for phage abundance and was subsequently normalized to the corresponding bacterial abundance within each metagenome. Phage abundances were expressed as the percentage of relative abundance to total bacterial abundances (Fig. S2).

## Results

### Occurrence of antimicrobial residues in the Göta Älv and associated WWTP effluents

**Figure 1:**
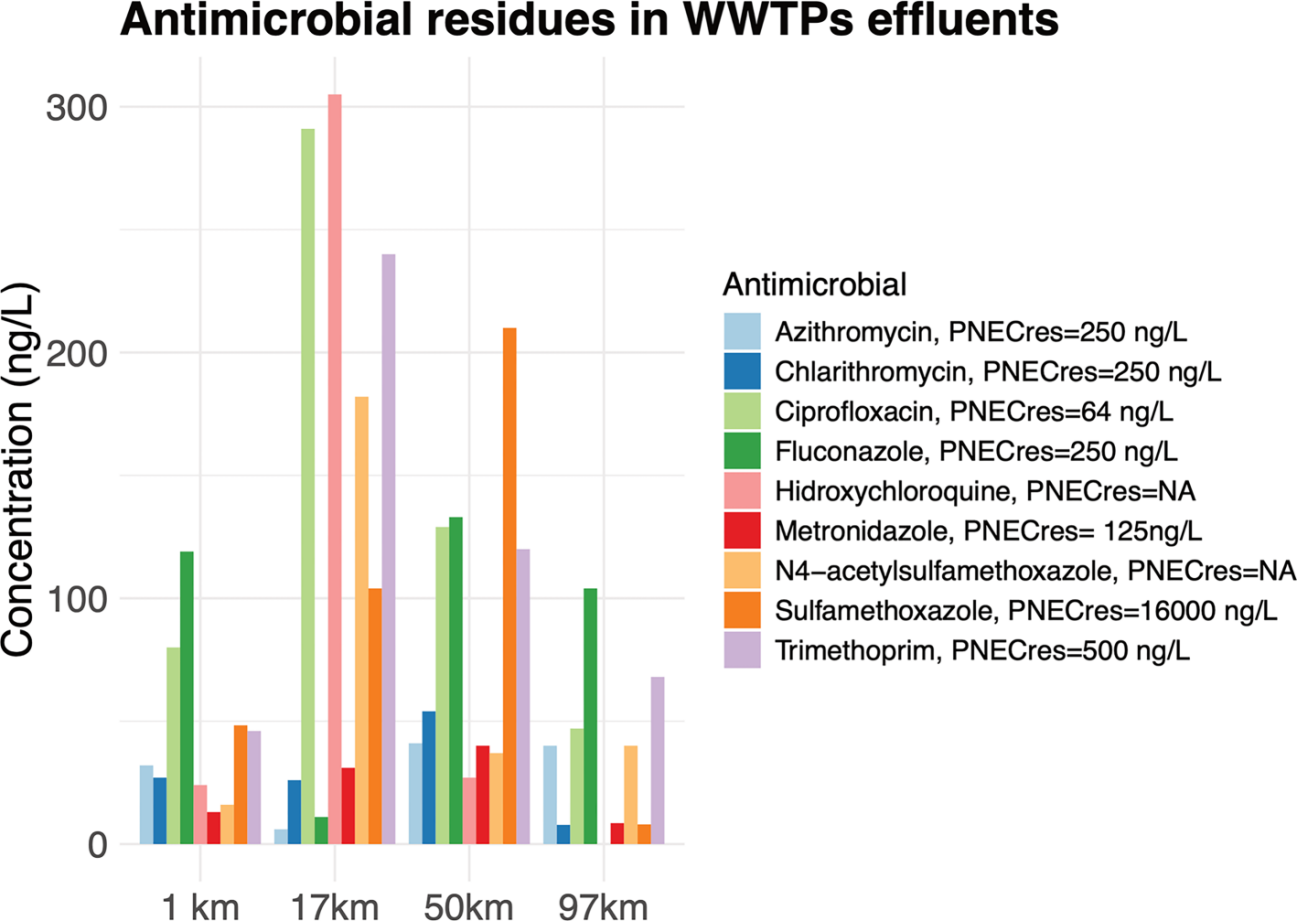
Concentration of antimicrobial compounds in effluents of the four WWTPs that discharge into the Göta Älv river (WWTP Vänersborg, WWTP Trollhättan, and WWTP Lilla Edet) and the Göta Älv estuary (WWTP Gothenburg). In the legend, the corresponding PNECres for AR development values for each antimicrobial are stated (Bengtsson-Palme and Larsson 2016). Concentration values are described in Table S5.

Out of the 30 antimicrobial residues analyzed, a total of 9 antimicrobials were recovered from the effluent samples from the WWTPs of all four municipalities above the quantification limits (Fig. 1; Table S5). The antimicrobials detected and their concentration ranges were as follows: azithromycin (6–41 ng/L), clarithromycin (7.8–54 ng/L), ciprofloxacin (47–291 ng/L), fluconazole (11–133 ng/L), hydroxychloroquine (24–305 ng/L), metronidazole (8.5–40 ng/L), N4-acetyl sulfamethoxazole (16–182 ng/L), sulfamethoxazole (8–210 ng/L), and trimethoprim (46–240 ng/L) (Fig. 1). Ciprofloxacin was present in the effluent water at concentrations above its predicted noneffect concentration (PNEC) for AR development (i.e. 64 ng/L; Bengtsson-Palme and Larsson 2016) in the WWTPs of Trollhättan, Lilla Edet, and Gothenburg. The rest of the screened antimicrobials were found to be below the minimum quantification limit (MQL) for effluent water (i.e. 2.5–470 ng/L). No antimicrobial residues were detected above their minimum quantification limit (MQL) in river surface water (i.e. 0.5–75 ng/L), except the fungicide fluconazole at a concentration of 0.95 ng/L on 18 km downstream of Trollhättan (Table S5).

### ARG profiling via qPCR in WWTP sludge, WWTP effluents, and river sediment

**Figure 2:**
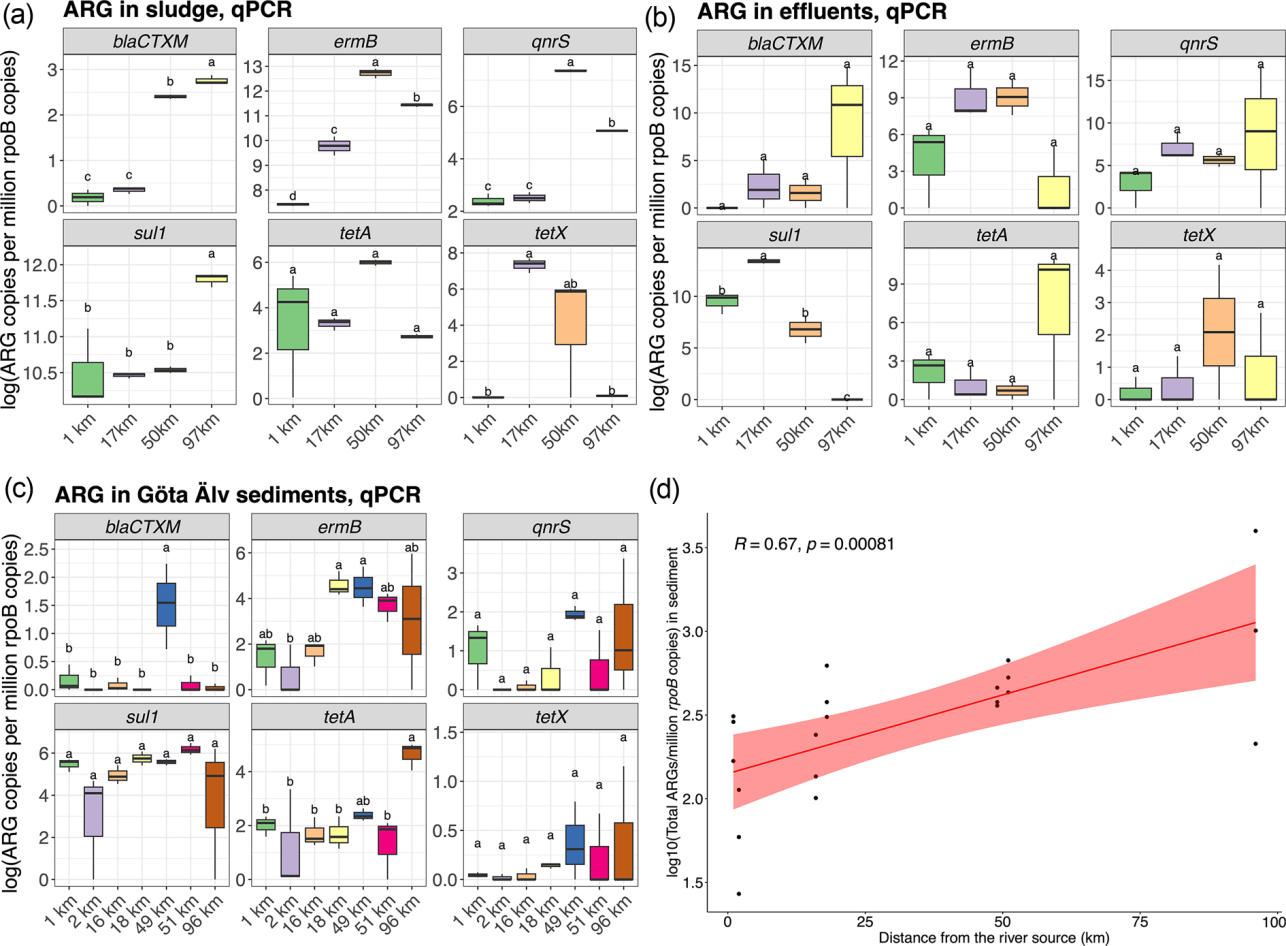
Abundance of the six qPCR-targeted ARGs (*bla*_CTX-M_*, ermB, qnrS, sul1, tetA*, and *tetX*) in the WWTP sludge (a), WWTP effluents (b), and Göta Älv sediments (c), expressed in the number of ARG copies per million *rpoB* gene copies. The different sampling sites along the Göta Älv river from the source (0 km) to the estuary (96 km) are represented in the *x*-axis (Fig. 2a–c). Colored boxes show the 25th–75th percentiles with the median (50th percentile) marked by the centre line. Lines indicate the 5th and 95th percentiles, and the displayed letters on top indicate a significant result between treatments in Tukey’s multiple comparisons post hoc test (*P* < 0.05). The results of Pearson correlation analysis between the distance from the river source and total ARG copies in sediment samples (d).

All genes targeted via qPCR were detected in all the sludges of the four WWTPs. The genes *ermB* (resistance to macrolides) and *sul1* (resistance to sulfonamides) were highly abundant in both the sludge and effluents of the four WWTPs compared to the other targeted genes (Fig. 2a and b). A higher presence of the genes *bla*_CTX-M_ (resistance to beta-lactams) and *qnrS* (resistance to quinolones) was observed in the sludges from WWTPs located along the river (50 and 97 km, *P*-adj*_bla_*_CTX-M_ = 3.12e − 09 and *P*-adj*_qnrS_* = 1.05e − 09), and *sul1* was more abundant in the WWTP sludge from the Gothenburg WWTP (97 km, *P*-adj*_sul1_*= 0.0009). The gene *tetX* was significantly more abundant in the sludges from WWTP located at 17 and 50 km along the river (*P*-adj*_tetX_* = 0.002), and *tetA* was equally abundant in all WWTPs (both conferring resistance to tetracyclines, *P*-adj*_tetA_* = 0.083). The different abundance patterns in the four sludges were not correlated to the number of users of each WWTP (Fig. S3.a; *P* = 0.12).

Gene abundance patterns detected in the sludges differed from their corresponding effluents (Fig. 2b), and gene abundances in the sludges were not correlated with the corresponding gene abundances in the effluents (Fig. S3b; *P* = 0.48). The genes *bla*_CTX-M_, *qnrS*, and *sul1* were detected at higher levels in the effluent samples than in their corresponding sludge samples. Moreover, the abundance of total genes in the effluent was not correlated with the number of users (Fig. S3c; *P* = 0.67).

Gene abundances in river sediments were 5000-fold lower than those in the effluent samples (Fig. 2c). The genes *sul1* and *ermB* were found at the highest relative abundances, following a similar trend as in the effluent and sludge samples. Abundances of *qnrS, sul1*, and *tetX* did not show statistical differences between river sites (*P*-adj*_qnrS_* = 0.092, *P*-adj*_sul1_* = 0.201, and *P*-adj*_tetX_* = 0.083). The gene *bla*_CTX-M_ showed a higher abundance peak at 49 km, upstream of the effluent of a WWTP (*P*-adj*_bla_*_CTX-M_= 0.0003), and the genes *ermB* and *tetA* showed a higher abundance trend downstream Göta Älv (from kilometer 49; *P*-adj*_ermB_*=0.0156, and *P*-adj*_tetA_*= 0.006). An increasing gradient in the total abundance of the six targeted ARGs from the river source to the mouth was observed (Fig. 2c) and was found to be positively correlated to the river distance from the source (*r* = 0.67; *P* = 0.00081, Fig. 2d), suggesting that the emissions of ARGs from the WWTPs contribute to the increase of ARGs in natural bacterial communities towards the river mouth.

### Resistome characterization via shotgun metagenomics in WWTP sludge, effluents, and river sediment

**Figure 3:**
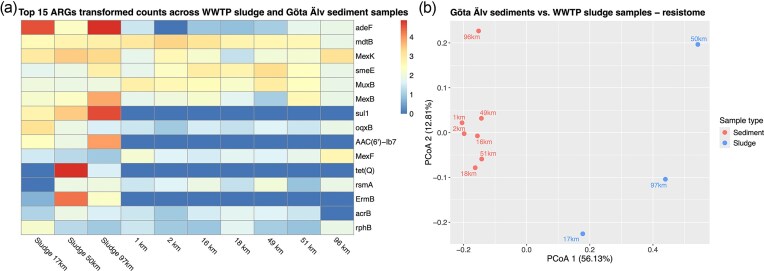
Resistome profile obtained from shotgun metagenomics analysis. Heatmap containing top 15 ARGs log-transformed counts across WWTP sludge and sediment samples (a), and PCoA plot based on Bray–Curtis distances, including sludge and sediment samples from the river source at 1 km to the river mouth at 96 km (b).

After the alignment of reads to the CARD database and filtering based on minimum sequence similarity and length, a total of 156 ARG were identified in at least one sample. The relative abundance of ARGs (counts per million of bacterial counts) was on average four times higher in sludge samples compared to the sediment samples (Fig. 3a). The genes *sul1* and *ermB* were among the top 15 genes found in WWTP sludge samples; however, they were not detected in river sediment samples. In the sludge, we observed a positive correlation between the relative abundances of *sul1* and *ermB* measured by qPCR and shotgun metagenomics (*r* = 0.91; *P* = 0.0002, Fig. S4). Genes belonging to the Mex-type multidrug resistance genes (*MexK, MexB*, and *MexF*) were among the top 15 most abundant genes in all samples (Fig. 3a). Principal coordinate analysis (PCoA) using the Bray–Curtis dissimilarity distances showed that the sludge resistome compositions differed from each other and from the sediment samples, respectively (PCoA1 = 56.13%; PCoA2 = 12.81%, Fig. 3b). All sediment samples were grouped together at the exclusion of the sludge samples, except the sample corresponding to the river mouth (96 km, Gothenburg), which differed from the rest (Fig. 3b). Moreover, the ARG abundance obtained in the sediments was not correlated with the distance from the river source, in contrast to the qPCR results (see section, ARG profiling via qPCR in WWTP sludge, WWTP effluents, and river sediment). Out of the 44 ARGs found in the river sediment samples, only one, *MexC*, was differentially abundant along the river gradient (adjusted *P*-value = 0.026).

**Figure 4:**
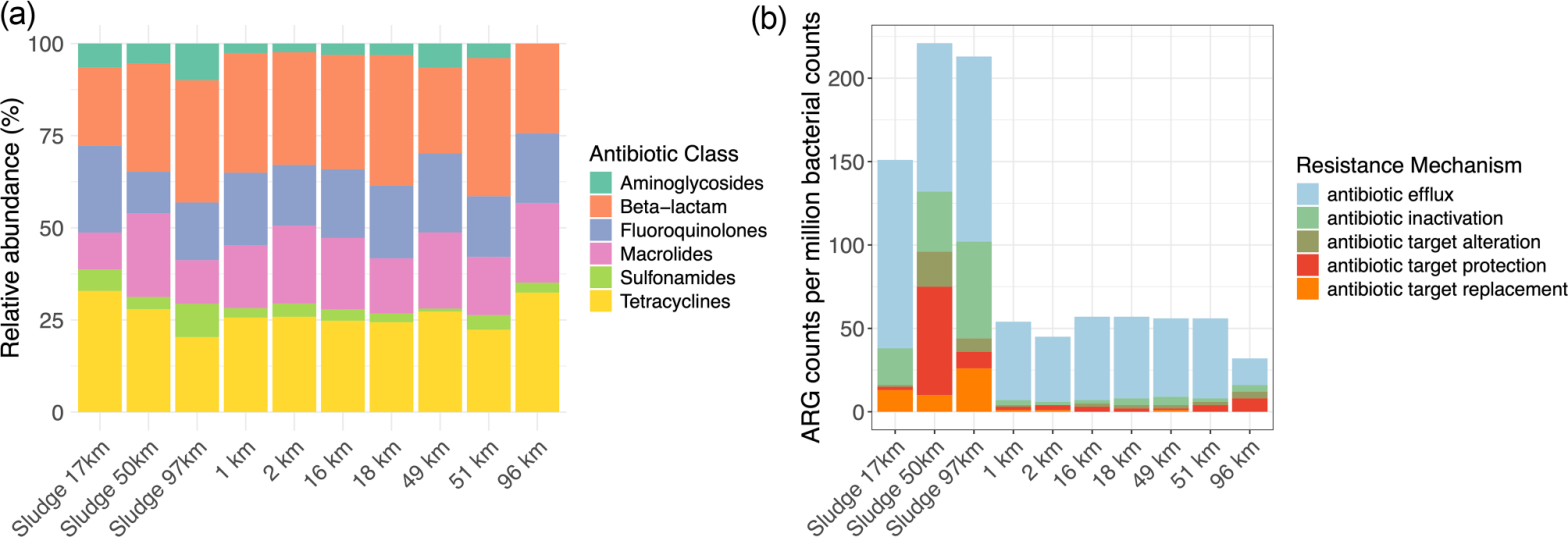
Relative abundance of resistance to antibiotic class of each resistome (a), and total counts per million bacterial counts grouped by resistance mechanism (b) for sludge and sediment samples.

In general, all sediment resistomes showed a similar pattern when grouped according to the main antibiotic class resistance (Fig. 4a). The ARGs corresponding to the downstream river sediment (96 km) did not show ARGs related to aminoglycoside resistance, while the rest of the sediment samples showed a relative abundance of between 2.3% and 6.6% to this group of antibiotics. The 96-km river sediment sample presented a higher relative abundance of resistance to tetracyclines (32.4%) compared to the rest of the sediment samples, which ranged from 22.3% to 25.8% (Fig. 4a). Interestingly, the sludge sample at 97 km showed the lowest relative abundance of resistance to tetracyclines compared to the other two sludges.

Efflux of antibiotics as a resistance mechanism presented the highest counts in all samples (Fig. 4b). The sludge at the WWTP located at 50 km of the Göta Älv showed the highest diversity of resistance mechanisms, followed by the sludge at 97 km. In contrast, the diversity of resistance mechanisms was lower among the sediment samples. The sediment in Gothenburg (96 km) contained a more diverse resistome in terms of resistance mechanisms, but the total ARG abundance was lower than in the rest of the sediment samples (Fig. 4b).

### Taxonomic profiling

**Figure 5:**
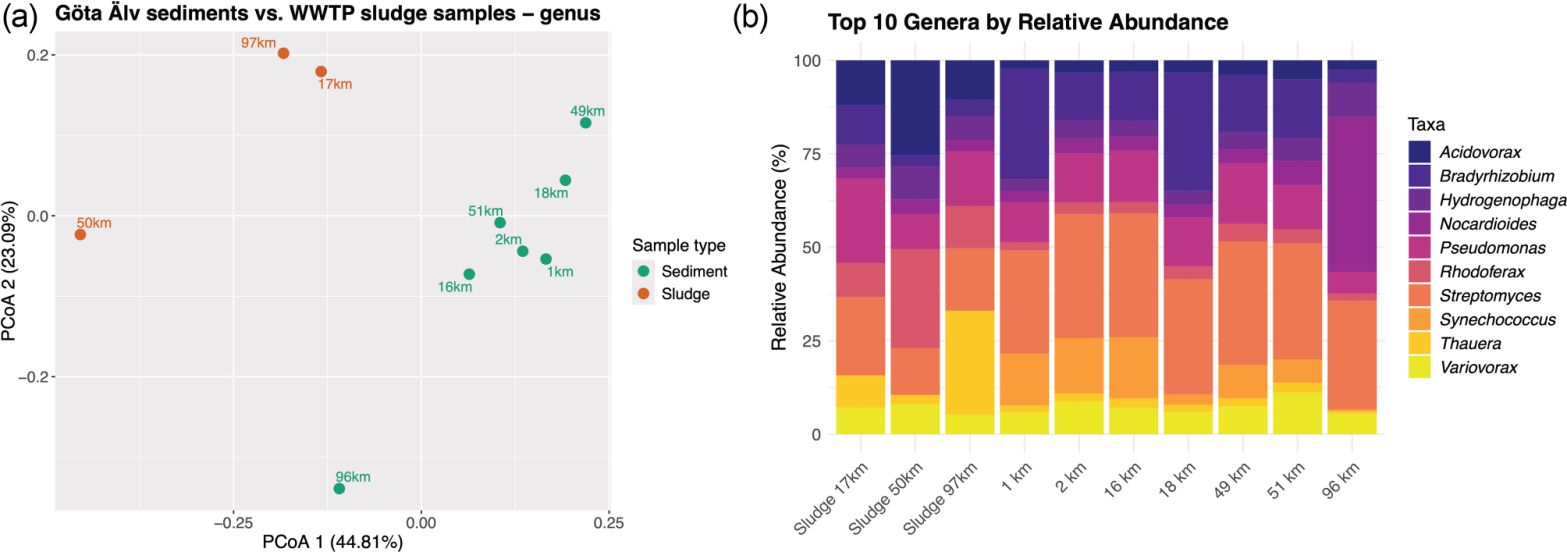
PCoA plot based on Bray–Curtis distances for genus beta-diversity, including sludge and sediment samples from the river source at 1 km to the river mouth at 96 km (a), and bacterial taxonomic profiles at the genus (b) level, resulting from prebuilt standard Kraken2 database alignments for all samples.

Shannon index values ranged between 4.14 and 4.66, and richness values ranged between 151 and 181 (Table 1 and Fig. S5). The Shannon index values in the sediment samples along the river were not correlated with the river distance (Fig. S6d). PCoA using Bray–Curtis distances revealed a similar grouping as the resistome (Fig. 5a). The genus-level diversity in the sludge at 50 km differed from the sludges at 97 and 17 km, and all the sludge samples differed from the sediment samples as well (PCoA1 = 44.81%; PCoA2 = 23.09%, Fig. 5a). As for the resistome composition (Fig. 3b), all sediment samples grouped when being compared to the sludge samples, except the sample corresponding to the river mouth (96 km, Gothenburg), which grouped far from the rest. However, the sludge diversities at the genus level (Shannon index, alpha diversity) did not show a correlation with the sludge resistome diversity or with the number of inhabitants connecting to the WWTPs (Fig. S6a and S6b).

A total of 323 different genera were found among all samples. Generally, all samples showed a similar relative abundance pattern with respect to the top 10 most abundant genera (Fig. 5b). The relative abundance of the genus *Streptomyces* was lower in sludge samples (3.18%–5.4%) compared to the sediments (9.55%–11.34%). The genus *Nocardioides* was more abundant in the sediment at the river mouth (96 km; 16.15%) than at any other river distance (Fig. 5b), and the genus *Pseudomonas* was among the most abundant genera in the sediment samples (2.3%–4.68%). However, the relative abundance of *Nocardioides* was not correlated with river distance (Fig. S6e).

All metagenomes were scanned for crAssphage (*Carjivirus communis*) presence as a gut bacteriophage indicator of human faecal pollution. The viral abundance was higher in the WWTP sludge samples compared to the river sediment (Fig. S2), with a maximum abundance in the sludge at 50 km (0.05% of normalized crAssphage coverage). The coverage in sediments ranged between 0.00013% and 0.0003%, with a maximum of 0.00031% at 18 km after the WWTP effluent (Fig. S2b). The crAssphage abundances in the river sediment did not follow a gradient pattern from upstream to downstream.

## Discussion

In this study, we characterized the resistome of sediment bacterial communities of the largest river in Sweden, Göta Älv, through two culture-independent techniques: qPCR and shotgun metagenomics. Our findings demonstrate that ARGs in river sediment increase along the length of the Göta Älv River and exhibit a different diversity from the upstream sites. While input from agriculture and other sources of bacteria carrying ARGs cannot be excluded, the increased abundance of target ARGs along the river was associated with ARGs released through WWTP effluents. Tetracyclines and beta-lactams were the most prevalent antibiotic classes to which ARGs present in the sediment-encoded resistance. Moreover, the genera *Pseudomonas, Streptomyces*, and *Nocardioides* dominated the microbiomes in river sediments. Finally, the sludge composition (resistome and microbial diversity, which was used as a reference since it is a concentrated reservoir of the ARGs within the WWTP) varied between WWTPs, and the number of WWTP users showed a positive correlation with the amount of ARGs released into receiving waters through the effluents.

Moreover, we analyzed the antimicrobial residues present in WWTP effluents and the receiving river water. Nine antimicrobial compounds were detected in all WWTP effluents, and ciprofloxacin reached concentrations up to 291.3 ng/L, being above its PNEC for AR development (i.e. 64 ng/L; Bengtsson-Palme and Larsson 2016). However, occurrence levels in surface water were below the quantification limit. Comparable values have been detected in other Scandinavian WWTP effluents (Sörengård et al. 2019). Among the identified compounds, only fluconazole was detected in surface river water at one sampling point (18 km) at a concentration of 0.95 ng/L. In Sweden, the median estimated national dilution factor of wastewater that would be diluted by the natural river flow is of 1825, which is considered high (>500) (see Johnson et al. (2020) and Keller et al. (2014)). The high dilution factor in the Göta Älv combined with the fact that ciprofloxacin was not detected in the river water samples suggests that river sediment bacteria in the Göta Älv are less likely to be exposed to concentrations of antibiotics that could lead to selective pressure, at least for the 30 antimicrobials we screened. Instead, the gradient enrichment of ARGs in the sediment along the Göta Älv is likely associated with ARG released from WWTP effluents, among other potential sources.

Of the six genes targeted via qPCR, the genes *sul1* and *ermB* were the most abundant in sludge, effluent, and sediment samples. Previous studies have reported resistance to sulfonamides (e.g. *sul1*) and macrolides (e.g. *ermB*) in river sediment (Brown et al. 2019, Ohore et al. 2019). Moreover, ARGs against sulfonamides (gene *sul1*) and β-lactams showed the highest average relative abundance compared to the rest of the studied genes in surface water from the rivers Eskilstunaån and Fyrisån, located on the Swedish east coast (Lai et al. 2021). The gene *sul1* has been identified as one of the most prevalent genes in the Vistula river (Poland) and has been previously found to represent the highest percentage of detected ARGs across various sampling sites in the Weihe river (China), including water, biofilm, and sediment (Harnisz et al. 2020, Li and Zhang 2020). In addition, the discharge of treated wastewater has previously been linked to a significant increase in the number of *sul1* gene copies in the sediment of the Warta river in Poland (Koczura et al. 2016). However, in our study, the different abundances of *sul1* gene copies in the effluents did not relate to changes in abundances of *sul1* in river sediment (Fig. 2b and c). Recently, Lee et al. (2021) reported that anthropogenic sources of ARGs decrease quickly over short distances (2–2.5 km) downstream of WWTP effluent discharge points. Regarding resistance to macrolides, the gene *ermB* has frequently been reported as an abundant gene in several rivers of the world (e.g. Adeniji et al. 2020, Proia et al. 2016, Rieke et al. 2018, Stoll et al. 2012, Su et al. 2014). Brown et al. (2019) reported *ermB* to be very abundant in a German river, reaching ∼10^6^ gene copies/100 ml in surface water samples.

The analysis of ARGs via qPCR also revealed a positive correlation between the total abundance of the six targeted genes and the distance from the river source. Our findings align with previous studies. For instance, Lai et al. (2021) found that ARGs detected through qPCR in urban recipient water bodies showed higher numbers of genes and relative abundances than upstream sites in Swedish rivers. Similarly, Chen et al. (2019a) observed a two-fold increase in total ARG abundance from upstream to downstream samples along the Chaobai river (China). These findings suggest that the transport of ARB and free DNA through the river course contributes to an increased abundance of ARG in downstream sections of human-impacted rivers. In addition, co-selection of AR bacteria due to chemical pollution (e.g. heavy metals) cannot be ignored (Gupta et al. 2022). The port of Gothenburg (estuary of the Göta Älv) is recognized to suffer from a significant contamination of water and sediment due to boat-associated pollutants such as Cu, Zn, and TBT (Norén et al. 2020). Therefore, co-selection or elevated rates of genetic exchange due to biofilm formations in the sediments could also be contributing to an increased abundance of ARG in the downstream sections of the river, as well as other unknown sources of ARGs. The absence of significant differences in gene abundances between upstream and downstream sites at each WWTP may be explained by the high dilution capacity of the river (the median dilution factor in Swedish rivers is 1825). Consequently, potential effects might become more evident further downstream, rather than at a distance of only 2 km from the WWTP outlet.

Our shotgun metagenomics analysis revealed that the genes *sul1* and *ermB* were among the most abundant of the top 15 genes identified in sludge samples. Although these genes were not detected in sediment samples through metagenomics, the number of *sul1* and *ermB* copies obtained via qPCR was positively correlated with the normalized number of the gene counts of *sul1* and *ermB* obtained in the metagenomic analysis. This further suggests that a gene should be present in thousands of copies per million bacterial cells (*rpoB* gene marker) detected with qPCR in order to be detectable through a metagenomics analysis, when normalized to total bacterial counts. Our study highlights the limitations and differences between qPCR and metagenomics: while *sul1* and *ermB* were detected in sediment samples through qPCR, shotgun metagenomics did not produce any counts, indicating that these genes were not among the most abundant in the Göta Älv’s sediments and highlighting the lower sensitivity of shotgun metagenomics compared to qPCR.

Moreover, shotgun metagenomics revealed the presence of multidrug resistance efflux pumps among the most frequently detected genes both in sludge and river sediments (e.g. Mex-type multidrug resistance genes) and indicated that the resistome and taxonomic profile at the river mouth (Gothenburg) differ from that of the upstream samples (Figs. 3b and 5b). The Mex-type multidrug resistance efflux pumps are the resistance nodulation division (RND) efflux pumps, which are responsible for antibiotic efflux and resistance in *Pseudomonas aeruginosa* (Langendonk et al. 2021). In our study, Mex-type genes were among the most abundant genes found in the sediment (i.e. *MexB* and *MexF*). They belong to the efflux pumps *MexAB-OprM* and *MexEF-OprN*, respectively, both of which are of clinical relevance (Alcalde-Rico et al. 2018). Together with *MexK*, these three genes were more abundant in the downstream section of the river (Figs. S7 and S8). The gene *MexC*, which was differentially expressed along the river, belongs to the efflux pump *MexCD-OprJ*, another clinically relevant efflux pump. Efflux pumps play a key role in AMR, although they comprise an intrinsic form of resistance (Puzari and Chetia, 2017). *MexCD–OprJ* does not contribute to AMR in wild-type *P. aeruginosa* under standard laboratory conditions (Purssell and Poole 2013). However, its overexpression in strains known as *nfxB* mutants leads to acquired multidrug resistance (Wang et al. 2024). The expression of *MexCD–OprJ* is solely regulated by the *NfxB* repressor, encoded by the *nfxB* gene, which was not detected in our study. The overexpression of *MexCD–OprJ* can be induced by membrane-damaging agents, including biocides, dyes, detergents, and organic solvents (Fraud et al. 2008, Purssell and Poole 2013). Our results indicate that the sediment at the river mouth, which is the most human-impacted area due to the proximity to the city of Gothenburg and its high boat traffic, harbors a different resistome diversity compared to the other sampling sites (Fig. 3a and b). Bacterial communities in this region would be expected to experience greater stress, and therefore would be more prone to overexpressing their RND efflux pumps, as has previously been found in other studies. However, a metatranscriptomic study would be necessary in order to determine if this is the case. Moreover, a higher abundance of RND efflux pumps might also be due to taxonomical shifts in the bacterial community.

The genus-level alpha diversity metrics (i.e. richness and the Shannon index) remained comparable across all samples, including sludge (Table 1). However, the beta-diversity in the sludge samples differed from each other, as did the sediment at the river mouth compared to the other sediments (Fig. 5b). Previous studies have shown that shifts in microbial diversity often coincide with changes in the resistome (Chen et al.2019b, Rubin-Blum et al. 2023, Samson et al. 2023). Moreover, Subirats et al. (2023) suggested that an increase in ARG efflux genes could provide a selective advantage in environments polluted with other chemicals, thereby inducing changes in the diversity of the microbiome.

The genera *Streptomyces* (9.55%–11.34%), *Pseudomonas* (2.3%–4.68%), and *Nocardioides* (0.99%–16.15%) were among the most prevalent genera in sediment samples. As mentioned previously, *Pseudomonas* is a carrier for Mex-type genes. The genus *Nocardioides* has been shown to exhibit a significant positive correlation with sulfonamide and tetracycline resistance genes (Chen et al.2019c, Guo et al. 2019). In our study, we found a higher abundance of *Nocardioides* in the estuarine region (river mouth). This genus is capable of utilizing a variety of organic substances as carbon sources, including petroleum hydrocarbons (Ma et al. 2023). The high presence of *Nocardioides* could be an indicator of environmental pollution, potentially linked to increased boat traffic and to the impact of other anthropogenic activities that contribute to the increase of ARG in the downstream sections of the Göta Älv.

## Conclusions

In this study, we characterized the presence and diversity of ARGs in the Göta Älv River sediments and associated WWTPs through qPCR and shotgun metagenomics. Our results showed that ARGs accumulate along the Göta Älv and exhibit a different diversity compared to the upstream sites, not only in terms of resistome composition but also at a taxonomic level. These differences may reflect the impact of WWTP effluents, in combination with other, yet unidentified, sources of ARGs and human activities. This study provided the opportunity to compare the two most commonly used DNA-based methods for ARG screening, demonstrating how they complement each other to offer a more integrative perspective. Overall, our findings improve our understanding of the spread and characteristics of ARGs in receiving urban rivers, which ultimately serve as raw water sources for humans and domestic animals that often rely on effective antibiotics.

## Supplementary Material

fiag007_Supplemental_Files
