# Supplementary material for: Antibiotic resistance gradient along a large Scandinavian river influenced by wastewater treatment plants
Source: FEMS Microbiol Ecol. 2026 Feb 3;102(3):fiag007. doi: 10.1093/femsec/fiag007 (PMC12917318; doi:10.1093/femsec/fiag007)
Supplement: fiag007_Supplemental_Files [file fiag007_supplemental_files.zip › supplementary_material_götaälv_revised.docx]

**Supplementary material**

**Antibiotic resistance gradient along a large Scandinavian river influenced by wastewater treatment plants**

**Supplementary Tables**

Table S1: Summary of the main characteristics of the four WWTPs that discharge in the Göta Älv river (WWTP Vänersborg, WWTP Trollhättan and WWTP Lilla Edet) and the Göta Älv estuary (WWTP Gothenburg) that were included in this study. See also Figure S1.

| WWTP (distance from river source) | Number of inhabitants connected to the WWTP | Type of users | Year of construction | Volume effluent water emitted into the River Göta Älv (m^3^/day) | GPS coordinates of WWTP outlet |
| --- | --- | --- | --- | --- | --- |
| Vänersborg (Km 1) | ~39 748 (2022) | Households | 1959 | ~15 000 | 58.384085, 12.349354 |
| Trollhättan  (Km 17) | ~59 154 (2021) | Households, industry, hospital | 1968 | ~30 070 (2021) | 58.124288, 12.121546 |
| Lilla Edet  (Km 50) | ~6 464 | Households | 1972 | ~2 745 | 58.123941, 12.121877 |
| Gothenburg  (Km 97) | ~887 442 (2021) | Households, industries, hospitals, and other activities | 1972 | ~3 416 216 (2021) m3/year | 57.690982, 11.890703 |

Table S2: GPS coordinates, general water quality parameters (Temperature, pH, conductivity, and concentration of PO_4_^3-^, NO_3_^2-^, and total organic carbon (TOC)) and chlorophyll-a concentration in grams of dry weight of sediment (Chl-a) for the seven sampling sites along the Göta Älv, from the source in lake Vänern (1 km) to Gothenburg estuary (96 km).

| Km | 1 | 2 | 16 | 18 | 49 | 51 | 96 |
| --- | --- | --- | --- | --- | --- | --- | --- |
| GPS coordinates | 58.384045, 12.341610 | 58.368927, 12.358567 | 58.288494, 12.282880 | 58.270413, 12.252951 | 58.125048, 12.120678 | 58.122457, 12.117423 | 57.694506, 11.903734 |
| Temperature (°C) | 11.9 | 12.1 | 12 | 12 | 11 | 11 | 10.8 |
| Conductivity (µS/cm) | 80 | 79 | 94 | 96 | 96 | 100 | 9728 |
| pH | 7.09 | 7.01 | 7.01 | 6.95 | 6.95 | 7.01 | 6.84 |
| PO_4_^3-^ (mg/L) | < 0.0050 | 0.006 | < 0.0050 | < 0.0050 | 0.01 | < 0.0050 | 0.007 |
| NO_3_^2-^ (mg/L) | 0.25 | 0.35 | 0.29 | 0.3 | 0.3 | 0.29 | < 0.1 |
| TOC (mg/L) | 4.6 | 4.6 | 8.1 | 8.3 | 11 | 8.5 | 12 |
| Chl-*a* (μg/gDW) | 18.17 | 13.77 | 21.52 | 11.93 | 1.88 | 1.78 | 2.43 |

Table S3: Target genes and primer sequences used in this study.

| Target gene | Gene type | Sequence (5' to 3') | Reference |
| --- | --- | --- | --- |
| *rpoB* | Reference | AACATCGGTTTGATCAAC CGTTGCATGTTGGTACCCAT | (Dahllöf et al., 2000) |
| *sul1* | Sulfonamide resistance | CGCACCGGAAACATCGCTGCAC TGAAGTTCCGCCGCAAGGCTCG | (Pei et al., 2006) |
| *tetA* | Tetracycline resistance | GTAATTCTGAGCACTGTCGC CTGCCTGGACAACATTGCTT | (Guardabassi et al., 2000) |
| *ermB* | Macrolide resistance | GATACCGTTTACGAAATTGG GAATCGAGACTTGAGTGTGC | (Chen et al., 2007) |
| *qnrS* | Quinolone resistance | GACGTGCTAACTTGCGTGAT TGGCATTGTTGGAAACTTG | (Marti and Balcazar, 2013) |
| *tetX* | Tetracycline resistance | CCGGAATTCAAGCTTTTATTATACATTTAACAATTGC CCGGAATTCCATATGACAATGCGAATAGATACAGAC | (Yang et al., 2004) |
| *blaCTX-M* | Beta-lactam resistance | CTATGGCACCACCAACGATA ACGGCTTTCTGCCTTAGGTT | (Proia et al., 2016) |

Table S4: qPCR optimization results for all target genes. SQ refers to starting quantity of DNA in nanograms.

| Target gene | qPCR conditions | Calibration curve | % Efficiency |
| --- | --- | --- | --- |
| *rpoB* | 98°C for 3 min (1 cycle); 98°C for 15 s, 50°C for 90 s, and 72°C for 90 s (30 cycles) 72°C for 10 min (1 cycle) | Cq=-3.27 log (SQ) + 2.3; R^2^ = 0.989 | 96.9% & 102.2% |
| *sul1* | 98°C for 3 min (1 cycle); 98°C for 15 s, 65°C for 30 s, and 72°C for 30 s (30 cycles) | Cq=-3.398 log (SQ) + 1.101; R^2^ = 0.995 | 93.5% & 96.9% |
| *tetA* | 98°C for 5 min (1 cycle); 98°C for 15 s, 62°C for 30 s, and 72°C for 45 s (40 cycles) 72°C for 7 min (1 cycle) | Cq=-3.55 log (SQ) + 0.786; R^2^ = 0.995 | 97.7% & 91.3% |
| *ermB* | 98°C for 3 min (1 cycle); 98°C for 15 s, 58°C for 30 s, 72°C for 30 s, and 78°C for 18 s (40 cycles) 95°C for 2 min, 55°C for 30 s, and 95°C for 30 s, (1 cycle) | Cq=-3.546 log (SQ) + 2.044; R^2^ = 0.997 | 103.3% & 91.4% |
| *qnrS* | 98°C for 3 min (1 cycle); 98°C for 15 s  and 65°C for 30 s (40 cycles) 95°C for 5 s | Cq=-3.389 log (SQ) + 1.232; R^2^ = 0.986 | 95% & 97.3% |
| *tetX* | 98°C for 3 min (1 cycle); 98°C for 30 s, 52°C for 60 s, and 72°C for 90 s (40 cycles) | Cq=-3.304 log (SQ) + 1.884; R^2^ = 0.996 | 103% & 100.8% |
| *blaCTX-M* | 98°C for 3 min (1 cycle); 98°C for 15 s  and 60°C for 30 s (40 cycles) 95°C for 5 s (1 cycle) | Cq=-3.299 log (SQ) + 0.616; R^2^ = 0.988 | 109.9% & 101% |

Table S5: Quantification of antimicrobials in WWTP effluent water samples collected in four WWTPs (Vänersborg, Trollhättan, Lilla Edet, and Gothenburg) and the seven sampling sites, in ng/L. The sampling was done from October 3-6, 2022. MQL = Method Quantification Limit.

*Excel file provided separately*

Table S6: DNA quality data. A260/280 and A 260/230 for each sequenced sample.

| **Sample ID** | **Sampling site** | **Eluent** | **A 260/280 ratio** | **A 260/230 ratio** | **DNA Preservation** |
| --- | --- | --- | --- | --- | --- |
| up_V_WWTP | upstream Vänersborg WWTP | DNAse free water | 1.94 | 1.79 | DNA stored at -20°C |
| dw_V_WWTP | downstream Vänersborg WWTP | DNAse free water | 1.95 | 1.28 | DNA stored at -20°C |
| up_T_WWTP | upstream Trollhättan WWTP | DNAse free water | 1.94 | 1.7 | DNA stored at -20°C |
| dw_T_WWTP | downstream Trollhättan WWTP | DNAse free water | 1.95 | 1.96 | DNA stored at -20°C |
| up_L_WWTP | upstream Lilla Edet WWTP | DNAse free water | 2.02 | 1.67 | DNA stored at -20°C |
| dw_L_WWTP | downstream Lilla Edet WWTP | DNAse free water | 1.93 | 1.8 | DNA stored at -20°C |
| up_G_WWTP | upstream Gothenburg WWTP | DNAse free water | 2.2 | 1.33 | DNA stored at -20°C |
| s_T_WWTP | sludge Trollhättan WWTP | DNAse free water | 1.92 | 2.1 | DNA stored at -20°C |
| s_L_WWTP | sludge Lilla Edet WWTP | DNAse free water | 1.91 | 2.19 | DNA stored at -20°C |
| s_G_WWTP | sludge Gothenburg WWTP | DNAse free water | 1.89 | 2.09 | DNA stored at -20°C |

**Supplementary Figures**


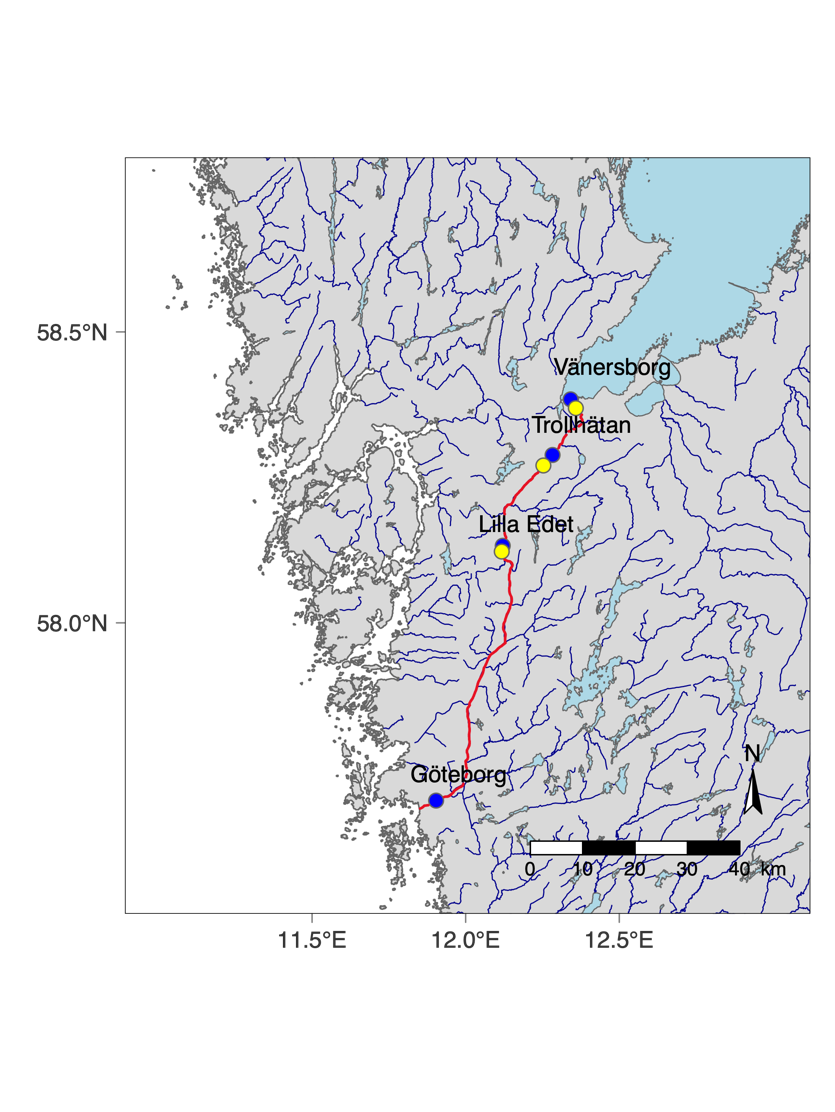


Figure S1: Map of the Göta Älv course (red), from lake Vänern in the northeast to the Kattegat Sea in the Atlantic to the west. Blue dots indicate river sediment sampling sites upstream of a WWTP, and yellow dots indicate river sediment sampling sites downstream of a WWTP.

a.

b.

Figure S2: Percentage of average crAssphage (indicator of human faecal pollution) genome coverages in relation to total bacterial abundances (%) in sludge (a) and river sediment (b).

a.

b.

c.

Figure S3: Pearson correlation analysis between total qPCR ARG copies in sludge and number of WWTP users (a), total ARG copies in effluents and sludge (b), and total number of ARGs copies in effluents and number of users (c).

Figure S4: Pearson correlation analysis between total number of ARG counts resulting from shotgun metagenomics and total number of copies obtained via qPCR in sludge.

Figure S5: Richness values in sludge (orange) and river sediments (green) along the Göta Älv, at genus level.

a.

b.

c.

d.

e.

f.

Figure S6: Pearson correlation analysis between Shannon index at genus level and Shannon index at resistome level in sludge (a), Shannon index at genus level in sludge and number of WWTP users (b), Shannon Index resistome in sediments and distance from the river source (c), Shannon Index at genus level in sediments and distance from the river source (d), relative abundance of *Nocardioides* and Shannon Index resistome (e), and total ARG copies per million bacterial counts in sediments and distance from the river source (f).

Figure S7: Heatmap containing top 15 ARGs log-transformed counts across sediment samples.

Figure S8: Heatmap containing Mex-type genes log-transformed counts across sediment samples.

a.

b.

Figure S9: Richness values in sludge (orange) and river sediments (green) along the Göta Älv, at genus level.

**Supplementary References**

1. Chen, J., Yu, Z., Michel, F. C., Jr, Wittum, T., & Morrison, M. (2007). Development and application of real-time PCR assays for quantification of erm genes conferring resistance to macrolides-lincosamides-streptogramin B in livestock manure and manure management systems. *Applied and environmental microbiology*, *73*(14), 4407–4416. https://doi.org/10.1128/AEM.02799-06
2. Dahllöf, I., Baillie, H., & Kjelleberg, S. (2000). rpoB-based microbial community analysis avoids limitations inherent in 16S rRNA gene intraspecies heterogeneity. *Applied and environmental microbiology*, *66*(8), 3376–3380. https://doi.org/10.1128/AEM.66.8.3376-3380.2000
3. Guardabassi, L., Dijkshoorn, L., Collard, J. M., Olsen, J. E., & Dalsgaard, A. (2000). Distribution and in-vitro transfer of tetracycline resistance determinants in clinical and aquatic Acinetobacter strains. *Journal of medical microbiology*, *49*(10), 929–936. https://doi.org/10.1099/0022-1317-49-10-929
4. Marti, E., & Balcázar, J. L. (2013). Real-Time PCR assays for quantification of qnr genes in environmental water samples and chicken feces. *Applied and environmental microbiology*, *79*(5), 1743–1745. https://doi.org/10.1128/AEM.03409-12
5. Pei, R., Kim, S. C., Carlson, K. H., & Pruden, A. (2006). Effect of river landscape on the sediment concentrations of antibiotics and corresponding antibiotic resistance genes (ARG). *Water research*, *40*(12), 2427–2435. https://doi.org/10.1016/j.watres.2006.04.017
6. Proia, L., von Schiller, D., Sànchez-Melsió, A., Sabater, S., Borrego, C. M., Rodríguez-Mozaz, S., & Balcázar, J. L. (2016). Occurrence and persistence of antibiotic resistance genes in river biofilms after wastewater inputs in small rivers. *Environmental pollution (Barking, Essex : 1987)*, *210*, 121–128. https://doi.org/10.1016/j.envpol.2015.11.035
7. Yang, W., Moore, I. F., Koteva, K. P., Bareich, D. C., Hughes, D. W., & Wright, G. D. (2004). TetX is a flavin-dependent monooxygenase conferring resistance to tetracycline antibiotics. *The Journal of biological chemistry*, *279*(50), 52346–52352. https://doi.org/10.1074/jbc.M409573200
